# Association Between Workplace Bullying Occurrence and post-traumatic stress disorder Among Healthcare Professionals in Tunisia

**DOI:** 10.1192/j.eurpsy.2022.1795

**Published:** 2022-09-01

**Authors:** M. Mnif, N. Smaoui, S. Omri, R. Feki, I. Gassara, M. Maalej, J. Ben Thabet, L. Zouari, N. Charfi, M. Maalej

**Affiliations:** Hedi Chaker University Hospital, Psychiatry C, Sfax, Tunisia

**Keywords:** Healthcare professionals, Post-traumatic stress disorder

## Abstract

**Introduction:**

Nurses may be particularly at risk of violence exposure at work which can cause psychological trauma and even develop post-traumatic stress disorder (PTSD), which is a serious mental health disorder.

**Objectives:**

The aim of this study was to determine the prevalence of PTSD among nurses in psychiatry and emergency departments and to identify the factors associated with it.

**Methods:**

This was a cross-sectional, descriptive and analytical study. It concerned 60 nurses working in the psychiatry (35 nurses) and emergency (25 nurses) departments of the Hedi Chaker and Habib Borguiba University Hospital in Sfax. The screening of PTSD was carried out by the « post-traumatic stress evaluation questionnaire » (PTSQ).

**Results:**

Direct trauma exposure was reported by 93% of participants, of which 48.3% experienced the act of violence more than 4 times. According to the PTSQ, 48.3% of the nurses had PTSD with a mean score of 50.93. Hyper-arousal was the most frequently observed outcome in victims (85%), followed by re-experience (83%) and avoidance (80%) symptoms. The presence of PTSD was correlated with female gender (p=0.002), the young age of the nurse (p=0.04), and the absence of peri-traumatic reactions (p=0.001).

**Conclusions:**

Our study shows that PTSD is a pathology frequently encountered in psychiatric and emergency nurses. Hence the need to put in place strategies against violence in hospitals and to apply them rigorously in order to better manage this phenomenon and manage its repercussions on health workers .

**Disclosure:**

No significant relationships.

